# Characterising risk of in-hospital mortality following cardiac arrest using machine learning: A retrospective international registry study

**DOI:** 10.1371/journal.pmed.1002709

**Published:** 2018-11-30

**Authors:** Shane Nanayakkara, Sam Fogarty, Michael Tremeer, Kelvin Ross, Brent Richards, Christoph Bergmeir, Sheng Xu, Dion Stub, Karen Smith, Mark Tacey, Danny Liew, David Pilcher, David M. Kaye

**Affiliations:** 1 Department of Cardiology, Alfred Hospital, Melbourne, Victoria, Australia; 2 Heart Failure Research Group, Baker Heart and Diabetes Institute, Melbourne, Victoria, Australia; 3 Department of Medicine, Nursing and Health Sciences, Monash University, Melbourne, Victoria, Australia; 4 Institute for Integrated and Intelligent Systems, Griffith University, Gold Coast, Queensland, Australia; 5 IntelliHQ, Gold Coast, Queensland, Australia; 6 Gold Coast University Hospital, Gold Coast, Queensland, Australia; 7 Faculty of Information Technology, Monash University, Melbourne, Victoria, Australia; 8 Centre for Research and Evaluation, Ambulance Victoria, Melbourne, Victoria, Australia; 9 Department of Epidemiology and Preventive Medicine, Monash University, Melbourne, Victoria, Australia; 10 School of Public Health and Preventive Medicine, Monash University, Melbourne, Victoria, Australia; 11 Department of Intensive Care, Alfred Hospital, Melbourne, Victoria, Australia; 12 Australian and New Zealand Intensive Care Society Centre for Outcome and Resource Evaluation, Melbourne, Victoria, Australia; Johns Hopkins University, UNITED STATES

## Abstract

**Background:**

Resuscitated cardiac arrest is associated with high mortality; however, the ability to estimate risk of adverse outcomes using existing illness severity scores is limited. Using in-hospital data available within the first 24 hours of admission, we aimed to develop more accurate models of risk prediction using both logistic regression (LR) and machine learning (ML) techniques, with a combination of demographic, physiologic, and biochemical information.

**Methods and findings:**

Patient-level data were extracted from the Australian and New Zealand Intensive Care Society (ANZICS) Adult Patient Database for patients who had experienced a cardiac arrest within 24 hours prior to admission to an intensive care unit (ICU) during the period January 2006 to December 2016. The primary outcome was in-hospital mortality. The models were trained and tested on a dataset (split 90:10) including age, lowest and highest physiologic variables during the first 24 hours, and key past medical history. LR and 5 ML approaches (gradient boosting machine [GBM], support vector classifier [SVC], random forest [RF], artificial neural network [ANN], and an ensemble) were compared to the APACHE III and Australian and New Zealand Risk of Death (ANZROD) predictions. In all, 39,566 patients from 186 ICUs were analysed. Mean (±SD) age was 61 ± 17 years; 65% were male. Overall in-hospital mortality was 45.5%. Models were evaluated in the test set. The APACHE III and ANZROD scores demonstrated good discrimination (area under the receiver operating characteristic curve [AUROC] = 0.80 [95% CI 0.79–0.82] and 0.81 [95% CI 0.8–0.82], respectively) and modest calibration (Brier score 0.19 for both), which was slightly improved by LR (AUROC = 0.82 [95% CI 0.81–0.83], DeLong test, *p <* 0.001). Discrimination was significantly improved using ML models (ensemble and GBM AUROCs = 0.87 [95% CI 0.86–0.88], DeLong test, *p <* 0.001), with an improvement in performance (Brier score reduction of 22%). Explainability models were created to assist in identifying the physiologic features that most contributed to an individual patient’s survival. Key limitations include the absence of pre-hospital data and absence of external validation.

**Conclusions:**

ML approaches significantly enhance predictive discrimination for mortality following cardiac arrest compared to existing illness severity scores and LR, without the use of pre-hospital data. The discriminative ability of these ML models requires validation in external cohorts to establish generalisability.

## Abstract 2

In their study, Shane Nanayakkara and colleagues find that machine learning-based models enhance predictive discrimination for mortality following cardiac arrest compared to existing approaches.

## Author summary

**Why was this study done?:**

- Cardiac arrest is a frequent cause of admission to the intensive care unit and has a low survival rate following admission to hospital.
- Current illness severity scores perform poorly in regard to predicting survival for this specific group of patients.
- Machine learning involves the creation of algorithms that can learn from large datasets to improve risk estimation, but can be biased by the data used.
- We aimed to use machine learning to predict death after admission to an intensive care unit with a cardiac arrest, and then to use an ‘explainer’ model to make the decision-making process transparent.

**What did the researchers do and find?:**

- We analysed one of the largest international datasets of patients admitted to the intensive care unit, comprising 1.5 million patients.
- We studied the data of patients admitted with cardiac arrest and developed several machine learning algorithms to predict death, and then compared these with existing scores.
- We found that the machine learning models were more accurate at estimating the risk of death, and were able to use another algorithm to explain the reasoning behind the risk estimate given for a particular patient.

**What do these findings mean?:**

- Using machine learning can increase the accuracy of estimating survival for intensive care patients after a cardiac arrest.
- These raw estimates can then be further resolved on a per-patient basis to provide a breakdown to understand the reasoning behind the algorithm’s decision, which could help clinicians decide whether to trust the algorithm on a per-patient basis.
- These findings have only been assessed in a single large group of patients, and should be validated in another separate group, with other predictors added.

## Introduction

Out-of-hospital cardiac arrest (OHCA) occurs annually in over 300,000 adults in the United States, with less than 11% of patients surviving to hospital discharge [1]. For those patients successfully resuscitated in the field, in-hospital mortality remains high, being accounted for by irreversible neurologic injury and by a post-cardiac arrest syndrome [2]. As a component of the clinical care of the OHCA patient, an assessment of the probability of survival after OHCA is performed to aid in the discussion between the clinical team and the patient’s family and to guide interventions. In this context, several prognostic tools have previously been developed. Several generic mortality prediction tools have been developed for intensive care unit (ICU) patients [3,4], however none specifically developed for this population after admission to the ICU. Such scores are valuable to benchmark outcomes between hospitals (particularly to interpret changes in outcomes over time in the context of illness severity); inform patients, families, and clinicians about prognosis; and identify subgroups that may be targets for interventions in trials, and compare intervention efficacy by allowing for appropriate baseline stratification.

In contrast to the above constrained approaches, machine learning (ML) describes the use of computer algorithms that learn non-linear associations retrospectively from the data to estimate the risk of a specified outcome. This method has been increasingly used in medical research recently for various purposes including image recognition [5–7] and patient phenotyping [8,9]. Outcome prediction has also been performed on a large scale with significant success through automated mining of electronic health records, combined with deep learning techniques [10]. Specifically in cardiovascular medicine, ML has been applied to imaging through echocardiography [11] and computed tomography [12], as well as outcome prediction in heart failure [13]. Although, as a whole, accuracy is gradually improving with ML techniques, a significant limitation has been the varying interpretability of certain models (particularly deep learning), and, due to their reliance on the data provided, such algorithms are heavily susceptible to bias [14]. The choice of algorithm is critical in providing a balance between interpretability and accuracy, although both of these terms are variably defined.

In this study, we utilised a large international registry [15] of intensive care admissions following cardiac arrest and applied a variety of ML methods to improve the prediction of outcomes, and compared these methods with logistic regression (LR) in addition to existing gold standard generic ICU illness severity scores. We hypothesized that ML techniques could predict early ICU mortality using basic demographic, physiologic, and biochemical data alone better than pre-existing illness severity scores.

## Methods

### Patient cohort

The Adult Patient Database is 1 of 4 clinical quality registries run by the Australian and New Zealand Intensive Care Society (ANZICS) Centre for Outcome and Resource Evaluation. Of the 214 ICUs in Australia and New Zealand, 186 have contributed data to the ANZICS Adult Patient Database [15]. The database presently collects data on 90% of all ICU admissions in Australia and New Zealand and contains information on over 2 million ICU episodes [16]. Institutional approval to undertake the study was provided by the Alfred Hospital Human Research Ethics Committee (Melbourne, Victoria, Australia), with a waiver of individual patient informed consent (Project No. 427/17). The study was commenced as part of the ANZICS Critical Care Datathon in March 2017 in Melbourne, Victoria, Australia.

### Variable selection

The following variables were extracted for ICU admissions between January 2006 and December 2016 (S1 Table): age, sex, comorbid conditions, and readmission status on admission to ICU; individual components of the Glasgow Coma Score prior to administration of sedation; urine output, highest and lowest physiologic and biochemical measures, and requirement for mechanical ventilation, all within the first 24 hours of ICU admission; and number of hours in the hospital prior to entering the ICU. The ANZICS database does not include continuous measurement variables, resulting in only the maximum and minimum measures being used. Electrocardiographic and echocardiographic data were not available. The APACHE III predicted risk of death—derived from a LR model developed from scoring the worst measurements over the first 24 hours of admission [17]—was included for comparison. The Australian and New Zealand Risk of Death (ANZROD) scoring system is the primary risk adjustment method for comparing mortality outcomes within Australia and New Zealand [18] and has been shown to provide better risk adjustment than the APACHE III scoring system [3], and so was also included for analysis.

### Patient selection

For this study, patients with an ICU admission diagnosis of cardiac arrest or who were listed as having had a cardiac arrest in the previous 24 hours prior to ICU admission were included. Primary diagnosis was determined using the ANZICS modification of the APACHE III diagnostic codes, determined at 24 hours post-admission by record review. Patients with elective admissions, those transferred from other ICUs, and those with treatment limitations were excluded. Patients with unknown mortality outcomes were also excluded. Mortality was determined as in-hospital death.

### ML models and pre-processing

Six algorithms were explored in this study: LR, random forest (RF), support vector classifier (SVC), gradient boosted machine (GBM), an ensemble approach, and an artificial neural network (ANN). These models are the most commonly used for binary classification problems in medicine, and we chose a wide selection to reflect this. In particular, GBMs often perform well with classification; however, an ensemble was included specifically to improve robustness when applied to an external dataset. Each model was supplied with the same input variables. Grid and random hyper-parameter searches were then used to search for optimal hyper-parameters for each model, with the area under the receiver operating characteristic curve (AUROC) as the optimisation metric. Upper and lower bounds for the hyper-parameters in the search were broad (such as decision tree depth between 2 and 100), so as to result in some underfitted models (with insufficient flexibility) and some overfitted models (which would not generalise due to excessive sensitivity to noise). Full hyper-parameter search ranges and final model hyper-parameters are available at the code repository online (https://github.com/IntelliHQ/CardiacArrestMortality_ANZICS). These models have been explained elsewhere in detail [19]; a brief summary is presented here. RFs utilise multiple decision trees to create a series of divisions in the data and generate an output. Decision trees choose these divisions based on maximising the decrease in impurity. GBMs are similar, involving a collection of weak decision models (in this case, decision trees), and combining these together through a process of iteratively training new models to address the weak points of the former models. SVCs aim to identify classes by creating a hyperplane of decision within a higher feature space in a non-linear fashion. The ANN uses a single hidden layer of neurons linking inputs to the output neuron, with weights trained using backpropagation and gradient descent to best approximate training data outputs [20]. The ensemble approach combined the RF, SVC, and GBM in a voting framework, where the individual algorithms each create a classification, and the most popular classification is taken to generate an overall prediction.

For missing value imputation, patients were separated into age group decades of life (<30, 30–39, 40–49, 50–59, 60–69, 70–79, ≥80 years), and missing values were imputed using the corresponding age group mean (for continuous variables) or mode (for categorical variables) for each variable. Continuous variables were standardised to a mean of 0 and variance of 1, and the dataset was split 90:10 into training and test sets. For training and tuning of the models, 5-fold cross-validation across the training set was used. Long term mortality was not available.

### Statistical methodology

We assessed model discrimination by calculating the AUROC. The Brier score (a measure of the mean squared difference between estimated risks and the actual outcomes) was calculated as a measure of model performance and calibration [21,22], and observed versus predicted plots are presented. Model accuracy was assessed using the logarithmic loss function.

To identify potential relevant features on a per-patient basis, we assessed explainability using local interpretable model-agnostic explanation (LIME). This method has been previously described in detail [23], and represents a well-validated model with robust code libraries. In brief, LIME generates a locally interpretable model for individual prediction from a complex model using an explainer algorithm that perturbs the inputs (in this case, the specific variables for a patient) together with an evaluation of the effects on the predictive model. This process generates a learned explanation for an individual.

All ML analyses were conducted using open-source software libraries (Python version 3.6.3, scikit-learn 0.19.1 [24], pandas [25], and H2O.ai), and visualisations and statistical analysis performed using R version 3.4.4 [26–28].

## Results

A total of 1,484,536 admissions to Australian and New Zealand ICUs were examined, of which 48,165 had a diagnosis of OHCA. After exclusions, there were 39,566 patients included for analysis, of whom 45.6% (18,019) did not survive to hospital discharge. Baseline characteristics of the patient cohort, categorised according to survival status, are presented in Table 1. Non-survivors were older (median 66 versus 63 years, *p <* 0.001), with a significantly higher peak creatinine (median 146 [IQR 102–198] versus 101 [76–151] μmol/l, *p <* 0.001). Non-survivors were more tachycardic (mean ± SD; peak heart rate 109 ± 26 versus 101 ± 23 bpm, *p <* 0.001) and slightly more hypotensive (lowest mean ± SD arterial pressure 61 ± 15 versus 66 ± 11 mm Hg). Of note, both maximum and minimum recorded temperature were similar between the 2 groups. There was a greater proportion of males among the survivors (66.2% versus 64.5%, *p <* 0.001).

**Table 1:** Demographic characteristics and physiologic parameters in the first 24 hours after out-of-hospital cardiac arrest.

Characteristic	Survivors (*n* = 21,547)	Non-survivors (*n* = 18,019)	*p-*Value
Age (years)	63 [50–73]	66 [52–77]	<0.001
Male sex	14,255 (66.2%)	11,629 (64.5%)	0.001
Intubated	15,846 (73.5%)	15,512 (86.1%)	<0.001
Highest temperature (Celsius)	37.05 (1.04)	36.75 (1.49)	<0.001
Lowest temperature (Celsius)	35.07 (1.47)	34.50 (1.60)	<0.001
Highest heart rate (bpm)	101 (23)	109 (26)	<0.001
Lowest heart rate (bpm)	65 (17)	69 (21)	<0.001
Highest respiratory rate (breaths per minute)	21 [18–25]	22 [18–27]	<0.001
Lowest respiratory rate (breaths per minute)	12 [10–14]	13 [12–15]	<0.001
Highest SBP (mm Hg)	146 (25)	142 (32)	<0.001
Lowest SBP (mm Hg)	94 (17)	87 (21)	<0.001
Highest DBP (mm Hg)	74 (15)	73 (18)	<0.001
Lowest DBP (mm Hg)	52 (10)	49 (13)	<0.001
Highest MAP (mm Hg)	99 (17)	96 (21)	<0.001
Lowest MAP (mm Hg)	66 (11)	61 (15)	<0.001
Highest sodium (mmol/l)	140 (4)	140 (5)	<0.001
Lowest sodium (mmol/l)	137 (4)	137 (5)	0.006
Highest potassium (mmol/l)	4.7 (0.8)	4.8 (0.9)	<0.001
Lowest potassium (mmol/l)	3.8 (0.6)	3.9 (0.7)	<0.001
Highest bicarbonate (mmol/l)	23 (4)	21 (5)	<0.001
Lowest bicarbonate (mmol/l)	20 (5)	17 (5)	<0.001
Highest creatinine (μmol/l)	101 [76–151]	146 [102–198]	<0.001
Lowest creatinine (μmol/l)	88 [65–126]	122 [86–150]	<0.001
Highest WCC (10^9^/l)	15.3 [11.3–19.4]	17.2 [13.0–22.0]	<0.001
Lowest WCC (10^9^/l)	12.0 [8.9–14.2]	13.1 [10.0–16.5]	<0.001
Highest platelet count (10^9^/l)	236 (85)	233 (92)	0.01
Lowest platelet count (10^9^/l)	199 (72)	195 (79)	<0.001
Highest glucose (mmol/l)	11.7 (5.3)	13.6 (6.2)	<0.001
Lowest glucose (mmol/l)	6.5 (2.3)	7.2 (3.8)	<0.001

Data are presented according to survival to hospital discharge. Parametric continuous variables are presented as mean (SD) and compared using Student *t* test; non-parametric variables are reported as median [IQR] and compared using the Wilcoxon signed rank test. Categorical variables are represented as number (percentage within category).

DBP, diastolic blood pressure; MAP, mean arterial pressure; SBP, systolic blood pressure; WCC, white cell count.

**Fig 1:**
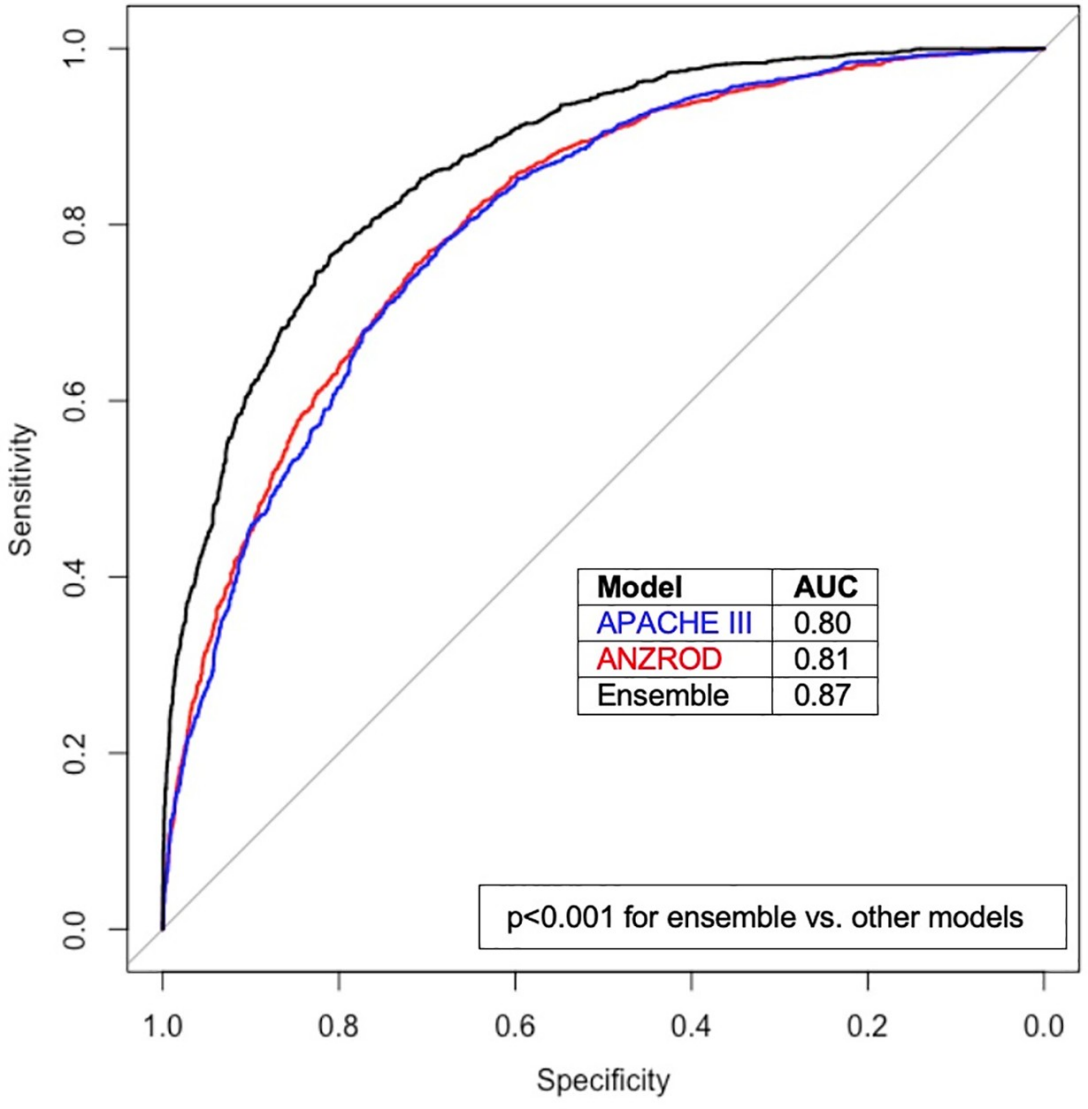
Receiver operating characteristic curves for existing prediction models and the ensemble ML model. ANZROD, red; APACHE III, blue; ensemble ML model, black. *p-*Value for comparison represents DeLong test. ANZROD, Australian and New Zealand Risk of Death; AUC, area under the curve; ML, machine learning.

Application of currently available illness severity models in the study population yielded an AUROC of 0.80 for the APACHE III score and 0.81 for ANZROD (Fig 1). Five ML models together with a LR model were tested and compared with the APACHE III and ANZROD scoring systems (Table 2).

**Table 2:** Performance of scoring systems and ML approaches for the estimation of in-hospital mortality in patients with an out-of-hospital cardiac arrest.

Model	Predicted mortality	AUC (95% CI)	Brier score	Log loss
Actual mortality	45.5%			
APACHE III risk of death	52.8%	0.80 (0.79–0.82)	0.190	0.57
ANZROD	39.9%	0.81 (0.80–0.82)	0.182	0.55
Logistic regression	45.4%	0.82 (0.81–0.83)	0.170	0.51
Artificial neural network	46.7%	0.85 (0.84–0.86)	0.158	0.48
Random forest	45.7%	0.86 (0.84–0.87)	0.156	0.47
Support vector classifier	45.4%	0.86 (0.85–0.87)	0.153	0.47
Ensemble	45.5%	0.87 (0.86–0.88)	0.148	0.45
Gradient boosted machine	45.3%	0.87 (0.86–0.88)	0.147	0.45

Results presented are based on test set (*n =* 3,957).

ANZROD, Australian and New Zealand Risk of Death; AUC, area under the curve; ML, machine learning.

### Statistical and ML models

LR outperformed both the APACHE III and ANZROD scoring systems, with a slightly higher AUROC (0.82, *p <* 0.01 using DeLong test for both comparisons; full parameter weights available at https://github.com/IntelliHQ/CardiacArrestMortality_ANZICS). Each of the ML models also attained a higher AUROC, with a very similar result between the GBM and the ensemble methods (AUROC = 0.87, *p* = 0.78). Brier scores were lower for LR and ML models, suggesting better calibration, with best results for the GBM and ensemble models (0.15 for both). Calibration plots are provided in S1 Fig.

**Fig 2:**
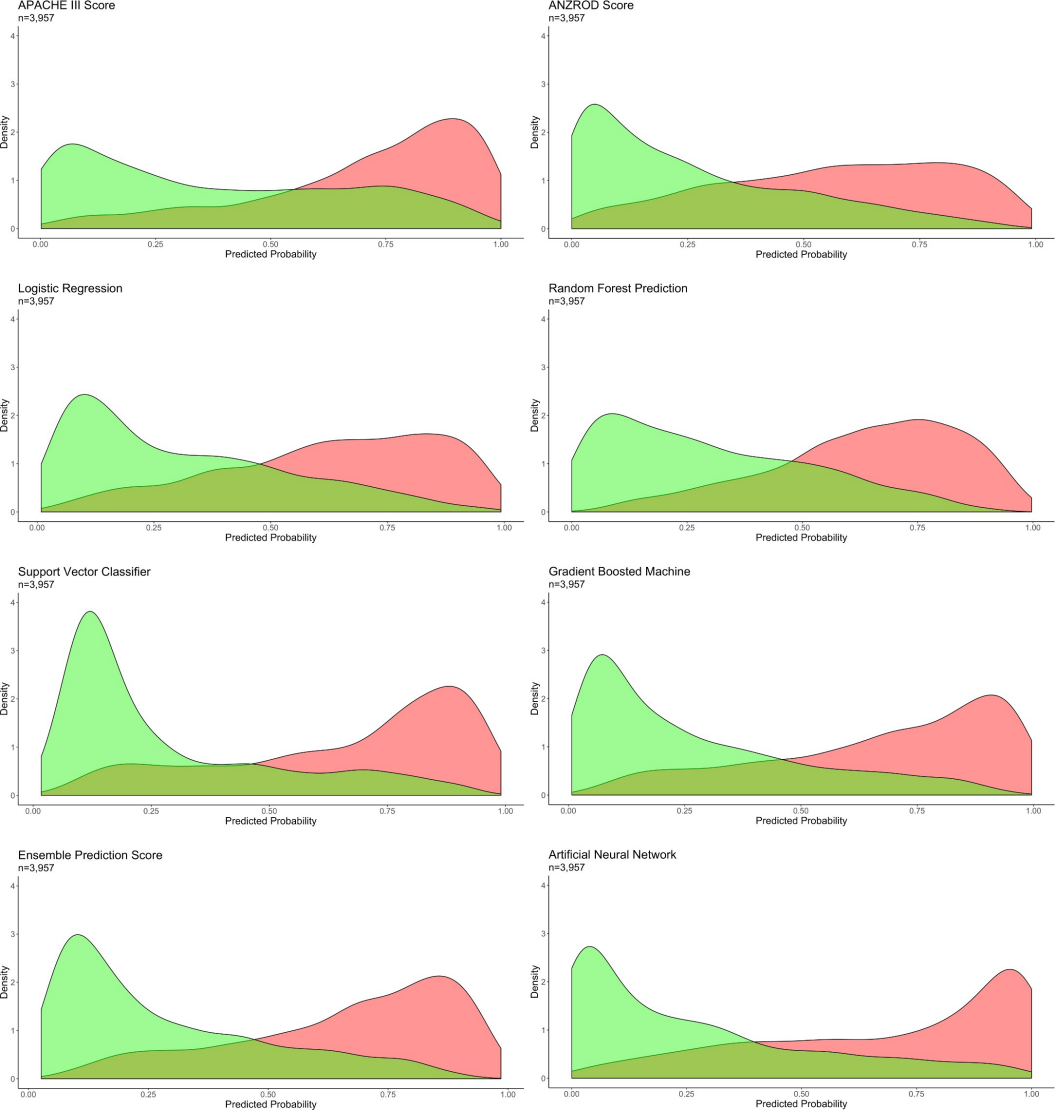
Probability curves for each model. Survivors indicated in green, and non-survivors in red. *p <* 0.001 for ensemble versus other models. ANZROD, Australian and New Zealand Risk of Death.

Probability curves were created for each of the models (Fig 2). Amongst non-survivors, the APACHE III score estimated a higher probability of death, with low variance in probability attributed to all survivors. The converse was apparent for the ANZROD score. The ML models—in particular the GBM, ANN, and ensemble models—demonstrated significant separation of the curves, with the lowest overlap in probability curves and greatest separation of the 2 groups.

**Fig 3:**
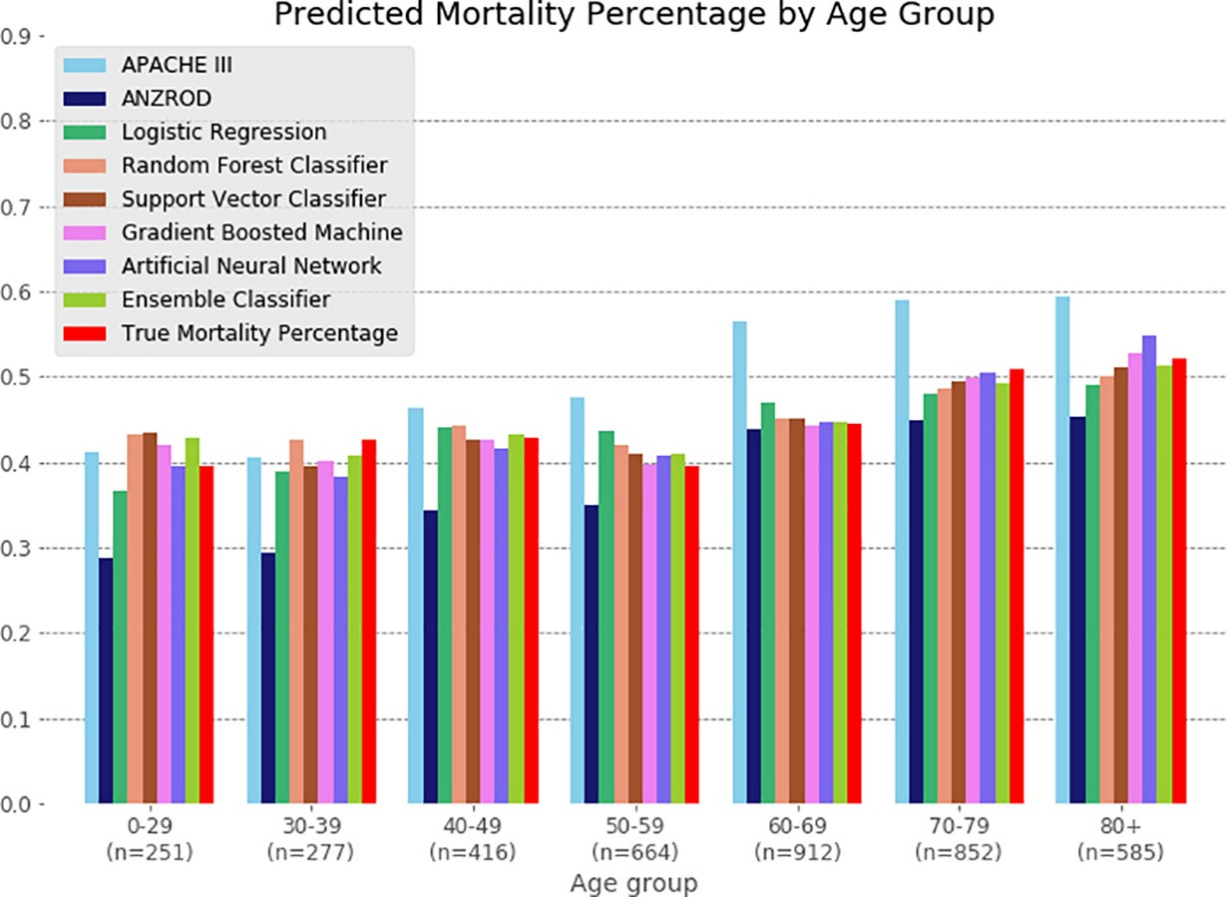
Sensitivity analysis comparing model performance across age groups. True mortality for each group is indicated in red. ANZROD, Australian and New Zealand Risk of Death.

Model comparisons with respect to mortality risk estimation were also performed across age groups (Fig 3). The APACHE model tended to overestimate mortality, particularly in patients over the age of 60 years, whereas the ANZROD model underestimated mortality in the youngest patients. The LR and ML models overall performed well.

### Model explainability

**Fig 4:**
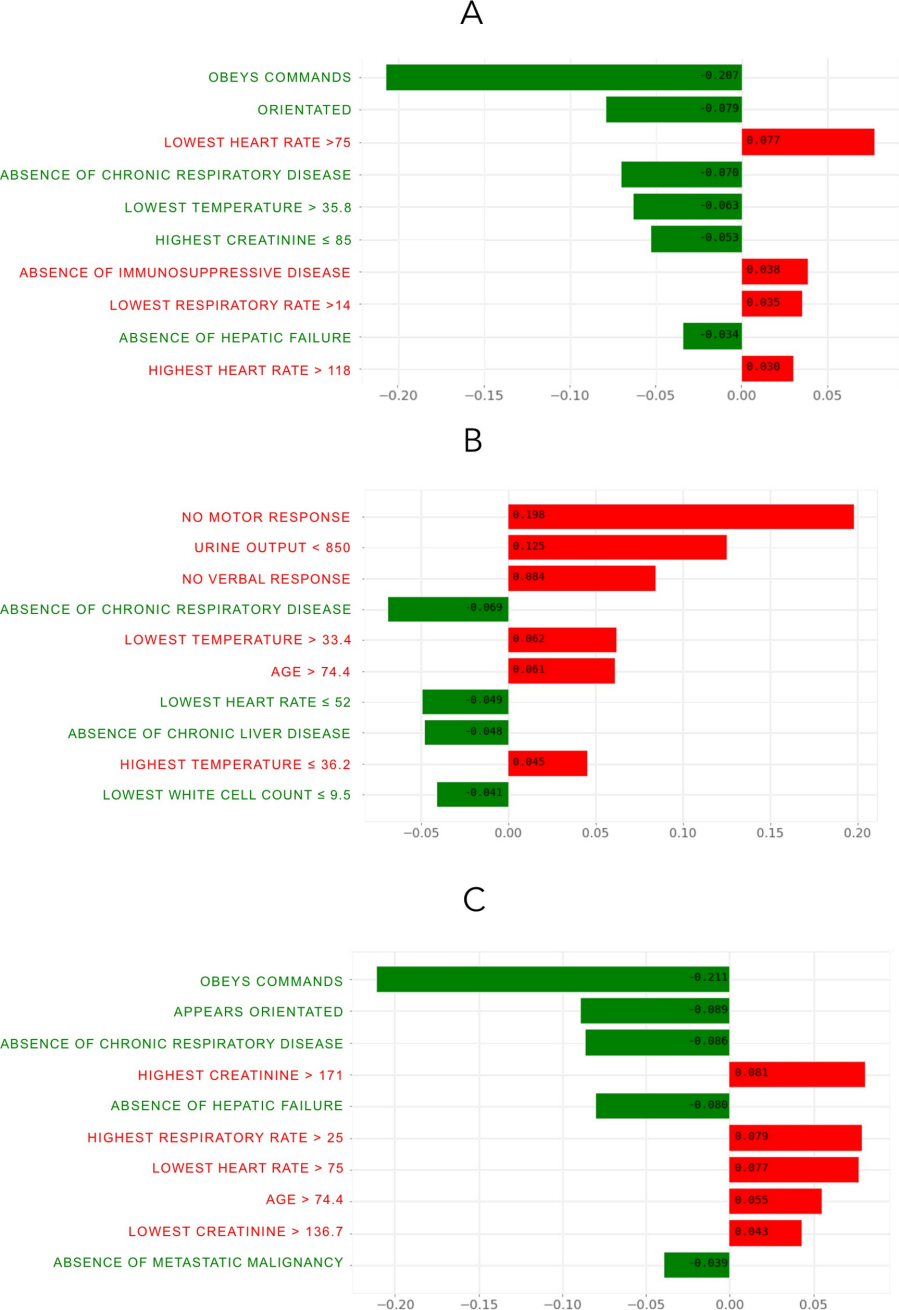
Local interpretable model explainer for 3 individual cases. (A) A correctly classified survivor, (B) a correctly classified non-survivor, and (C) an incorrectly classified non-survivor (predicted to survive). Features with a green bar favoured survival, and those with a red bar were predictive of mortality. The *x*-axis shows how much each feature added or subtracted to the final probability value for the patient (i.e., a feature with a weight of 0.2 is equivalent to a 20% change in the probability of survival).

We next applied the LIME explainer model to data generated by the ensemble ML model, particularly to explore misclassification. Cases with high and low predicted mortality rates were compared. The top 10 features for each case are presented in Fig 4, with the weight of each feature represented in either green or red depending on whether it favoured survival or death. Each weight can be interpreted in the context of the original probability; if a feature was absent for a patient, it can be numerically added to or subtracted directly from the initial probability. In the first correctly predicted case (Fig 4A), we show a specific individual with a high probability of survival (83%). The high scores for the motor (~21% impact favouring survival) and verbal components of the Glasgow Coma Score, absence of chronic respiratory disease, absence of hypothermia, and relatively preserved creatinine were all favourable; conversely, although this patient survived, negative prognostic factors included a minimum heart rate over 75 bpm (8% increased probability of death) and lowest respiratory rate over 14 breaths per minute.

In the second case (Fig 4B), again correctly predicted, the predicted probability of survival was 27%. The explainer algorithm notes that the lack of a motor response (20% increased probability of death), low urine output (less than 850 ml in 24 hours, 13% increased probability of death), hypothermia, and higher age were all markedly negative prognostic factors, with the lack of respiratory and hepatic disease and the presence of bradycardia being protective.

The explainer was then applied to incorrectly predicted patients. In the case presented in Fig 4C, the patient was attributed a survival probability of 78% however did not survive. Favourable features such as intact neurologic status and lack of chronic respiratory disease or hepatic failure led the algorithm toward survival; however, the explainer notes that the markedly elevated creatinine (>171 μmol/l), tachypnoea, and higher age (82 years) were all negative prognostic factors.

## Discussion

In this study, we demonstrated that modern ML approaches using physiologic and biochemical data collected during the first 24 hours after hospital admission for OHCA provide superior predictive capacity compared to existing illness severity models. To date, established tools such as the APACHE score have been used to provide prognostic information in critically unwell patients. In the current study, we showed that while these tools performed well in OHCA, they were less accurate than ML methods.

### Mortality prediction in cardiac arrest

In cardiac arrest, pre-hospital data are useful to explain much of the variation in survival to discharge [1], particularly in regard to factors such as witnessed cardiac arrest, initial rhythm, and bystander CPR. The OHCA score [29], a multivariate LR model developed on 130 patients, achieved an AUROC of 0.82 (0.88 in the validation cohort), although the sample size was small and there was significant class imbalance. Furthermore, the OHCA model requires knowledge of the periods of time with circulatory no flow and low flow, limiting its use to when pre-hospital data are known. Follow-up validation in a 173-patient cohort treated with therapeutic hypothermia demonstrated lower discrimination (AUROC 0.74), although the OHCA score still outperformed an existing illness severity score (SAPS II at 48 hours; AUROC 0.72) [4]. In another 21-variable LR model, an AUROC of 0.83 was obtained, with key predictors being pre-hospital variables (number of minutes to sustained restoration of spontaneous circulation and first rhythm) [30]. Biomarkers in smaller studies have also shown promise [31].

The accurate assessment of prognosis in OHCA patients is important for several reasons. In an effort to evaluate new therapeutic tools, the ability to select similar risk groups would be of utility in clinical trial design. From the perspective of healthcare utilisation, early identification of patients with a high risk prognosis may better assist in the timing of relevant changes in the clinical treatment objectives. Finally, for information dissemination to family and friends of the patient, the provision of an accurate estimation of prognosis is important.

This study differs from previous work in several domains. First, training ML models requires large amounts of well-curated data, whereas smaller datasets are inherently more prone to bias [14]. In this study, we used one of the largest intensive care databases in the world to generate our cardiac arrest subset. Second, a traditional strength of ML models is the ability to combine a large and diverse array of variables that often need to be entered manually, which may be laborious and impractical; in this study we were able to use maximum and minimum variables available at 24 hours after ICU admission only, with no pre-hospital data, and improve accuracy beyond existing models. This study extends previous work in outcome prediction in cardiac arrest, where models have been limited in predictive accuracy or have required vast amounts of pre-hospital data. Furthermore, this work extends further to add patient-level explanations to the ML approaches.

### Explainability

**Fig 5:**
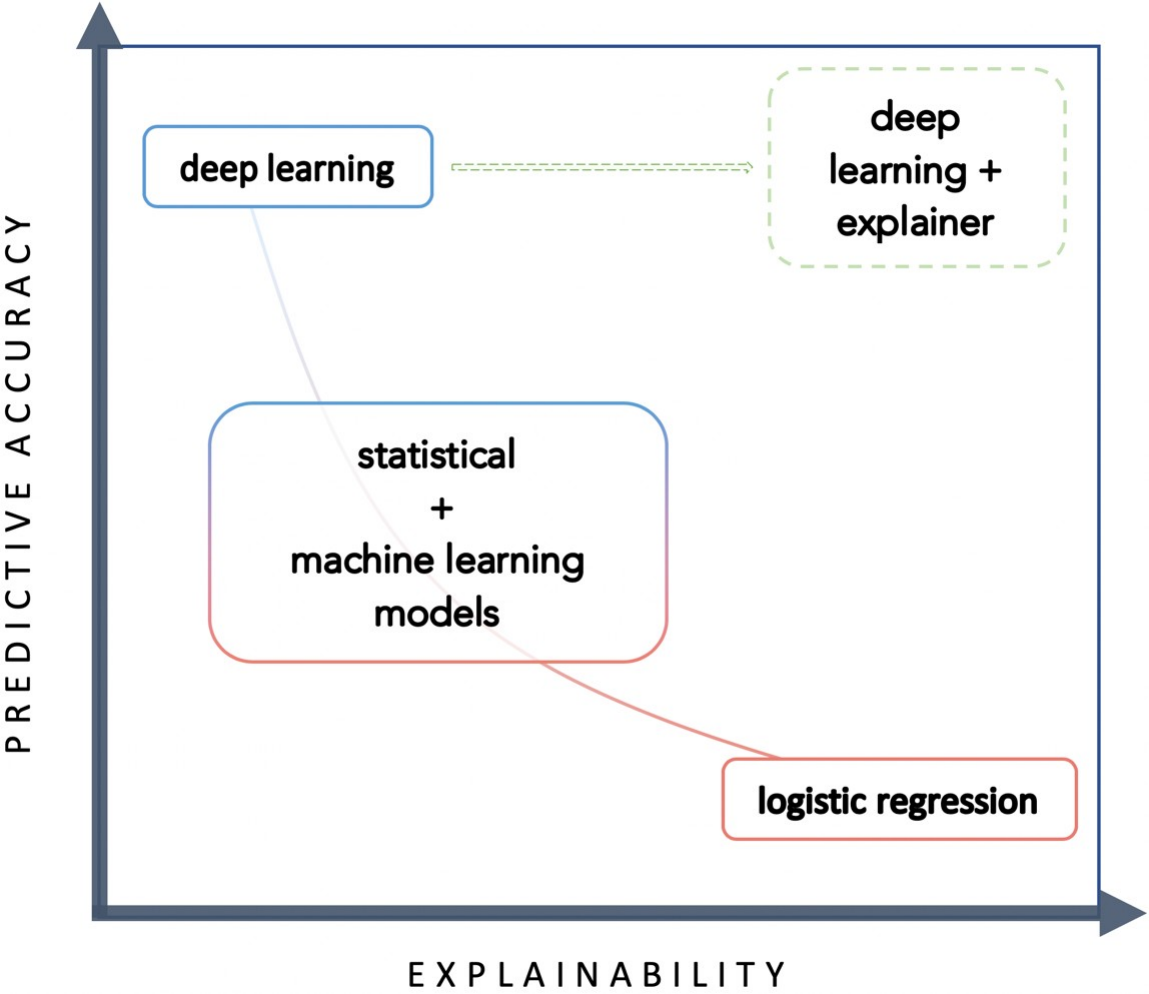
Trade-off between predictive accuracy and explainability.

Traditionally, there has been an inherent trade-off between accuracy and interpretability in modelling (Fig 5); simpler statistical models such as regression techniques have provided easy-to-understand models with significant heterogeneity in accuracy, while ML models have demonstrated remarkable accuracy with reduced interpretability, such that these models are often deemed to be ‘black boxes’. Increasing attention is being paid to the explainability of ML algorithms [23,32].

At this time, explainer models are not provided to provide a change to therapeutic choices; rather, explainer models may be used to understand how an algorithm came to its conclusion. Broadly speaking, interpretability enhances the model by increasing transparency—can a model’s decisions be trusted? Interpretability may assist the user in recognising data bias; however, it does not prevent it, as bias is fundamentally dependent on the quality and breadth of the data used to generate the algorithm.

By understanding a model’s predictive reasoning, researchers and clinicians can begin to explore whether the model’s decisions were made based on biases within the data (inevitable, particularly in registry-based data) by reflecting on their own clinical judgement. These biases can then be specifically targeted to improve the model. If augmented decision-making is to occur through the implantation of these algorithms, then the imperative is on the algorithm controller to provide meaningful information about the logic involved, and the consequences of predictions must be clearly understood. Of note, the recent General Data Protection Regulation applied in Europe has clearly stated that individuals who have decisions made about them by algorithms have a right to know the basis of the decision and the factors that influenced this in the predictive model.

Put simply, explainer algorithms significantly improve understanding of how algorithms arrive at a decision (such as through visualisation with LIME), allowing clinicians to then interpret whether they agree with the methodology before interpreting the conclusion. Recent landmark papers have incorporated explainability for this reason [7].

### Limitations

First, despite the large size of the dataset we used (~1.5 million records), the cardiac arrest subset was relatively small (*n =* 39,566), and as such these methods warrant repeating in a larger dataset. Second, as pre-hospital variables play a major prognostic role, the inclusion of pre-hospital observations may markedly improve accuracy and provide a better explanation for poor outcomes. By definition, the model already incorporates the end result of interventions prior to the time at which observations or measurements were made. Accordingly, the model includes a vector component triggered by an earlier intervention. Third, we recognise that this dataset pertains to the Australian and New Zealand population, both in regard to patient and system characteristics, and that our findings may not be applicable to other jurisdictions. Other intensive care datasets such as MIMIC-III [33] and eICU (from the US), together with specific cardiac arrest datasets (such as INTCAR or CARES), provide further opportunities for validation, particularly as they include all variables across the time of admission, rather than just highest and lowest physiologic measures. Imputation in this dataset was performed using age categories, relevant to cardiac arrest specifically, but more advanced imputation techniques such as chained equations should be considered in future work. Our dataset also had no information on therapies or on end-of-life practices or preferences, and changes in practice over time and their impact on outcomes were not assessed.

### Clinical translation

Accurate prediction models allow for improved clinician prognostication, better risk adjustment and hospital benchmarking, earlier identification of outlier centres, and improved patient–physician–family communication. There are multiple ways to report model performance; good discrimination suggests that a model separates survivors from non-survivors well, while good calibration refers to the agreement between the outcomes and the predictors—both are necessary for clinical translation of predictive models.

To improve clinical utility, prognostic scoring systems would ideally use data immediately available at the time of decision-making. Models such as those demonstrated here could be meaningfully used to perform risk adjustment between hospitals and, where available, pre-hospital data could be added to a trained GBM or ensemble model to produce accurate comparator data with respect to the management of cardiac arrest. Similarly, if models are being used for early prognostication, then both pre-hospital and in-hospital data up to the point of decision should be utilised. In this study, variables collected reflected the highest and lowest values for the first 24 hours of inpatient admission, with no pre-hospital data included. By using in-hospital data and basic demographic variables only, there is the potential for the algorithms shown here to be automated, deriving variables from the electronic health records of the facility at the end of the 24-hour period following admission. Finally, a key component of acceptance of ML models in practice revolves around explainability. Fundamentally, medical decision-making is based on a trust of the data provided, and in view of the potential consequences of medical decisions, understanding the reasoning behind predictions is essential.

Clinicians are unlikely to blindly trust an algorithm that is not both well validated and easily explainable. Explainer models can provide evidence of the machine’s thought process to arrive at the final prediction conclusion, which in the first instance can allow the clinician to determine whether the conclusion should be believed at all. Datasets will grow richer, and explanations more complex, as algorithms form part of the electronic medical record, rather than requiring manual data entry post hoc; this integration could potentially create new ‘biomarkers’ calculated frequently to alert clinicians to clinical deterioration. Eventually, well-explained predictions could be linked to physiologic pathways (through a process of phenomapping) to focus targeted therapies and improve patient outcomes.

### Conclusion

ML models based only on data from the first 24 hours of patient admission after cardiac arrest significantly improve the accuracy of prediction for in-hospital mortality, compared with existing illness severity scores. Explainer models provide patient-level explanations for ML predictions, for clinician interpretation of accuracy. These findings may improve individual prognostication, assist information provision, and prove useful for hospital-level risk adjustment in regard to the management of cardiac arrest.

## Supporting information

S1 Fig Calibration plots for all models. (DOCX)

S2 Fig Missing data for each variable prior to imputation. (PNG)

S1 Table Variables included in the LR and ML models. (DOCX)

S2 Table Comparison of performance metrics calculated on the test set, based on optimal thresholds (maximising sensitivity and specificity) obtained from receiver operating characteristic curves. (DOCX)

S1 TRIPOD TRIPOD checklist. (DOCX)

## Abbreviations

ANN: artificial neural network
ANZICS: Australian and New Zealand Intensive Care Society
ANZROD: Australian and New Zealand Risk of Death
AUROC: area under the receiver operating characteristic curve
GBM: gradient boosted machine
GCS: Glasgow Coma Score
ICU: intensive care unit
LIME: local interpretable model-agnostic explanation
LR: logistic regression
ML: machine learning
OHCA: out-of-hospital cardiac arrest
RF: random forest
SVC: support vector classifier

## References

[1] Benjamin EJ, Virani SS, Callaway CW, Chang AR, Cheng S, Chiuve SE, et al Heart disease and stroke statistics—2018 update: a report from the American Heart Association. Circulation. 2018;137:e67–492. 10.1161/CIR.0000000000000558 29386200 10.1161/CIR.0000000000000558

[2] Stub D, Bernard S, Duffy SJ, Kaye DM. Post cardiac arrest syndrome: a review of therapeutic strategies. Circulation. 2011;123:1428–35. 10.1161/CIRCULATIONAHA.110.988725 21464058 10.1161/CIRCULATIONAHA.110.988725

[3] Paul E, Bailey M, Pilcher D. Risk prediction of hospital mortality for adult patients admitted to Australian and New Zealand intensive care units: development and validation of the Australian and New Zealand Risk of Death model. J Crit Care. 2013;28:935–41. 10.1016/j.jcrc.2013.07.058 24074958 10.1016/j.jcrc.2013.07.058

[4] Choi JY, Jang JH, Lim YS, Jang JY, Lee G, Yang HJ, et al Performance on the APACHE II, SAPS II, SOFA and the OHCA score of post-cardiac arrest patients treated with therapeutic hypothermia. PLoS ONE. 2018;13:e0196197 10.1371/journal.pone.0196197 29723201 10.1371/journal.pone.0196197 PMC5933749

[5] Esteva A, Kuprel B, Novoa RA, Ko J, Swetter SM, Blau HM, et al Dermatologist-level classification of skin cancer with deep neural networks. Nature. 2017;542:115–8. 10.1038/nature21056 28117445 10.1038/nature21056 PMC8382232

[6] Narula S, Shameer K, Salem Omar AM, Dudley JT, Sengupta PP. Machine-learning algorithms to automate morphological and functional assessments in 2D echocardiography. J Am Coll Cardiol. 2016;68:2287–95. 10.1016/j.jacc.2016.08.062 27884247 10.1016/j.jacc.2016.08.062

[7] De Fauw J, Ledsam JR, Romera-Paredes B, Nikolov S, Tomasev N, Blackwell S, et al Clinically applicable deep learning for diagnosis and referral in retinal disease. Nat Med. 2018;24:1342–50. 10.1038/s41591-018-0107-6 30104768 10.1038/s41591-018-0107-6

[8] Gehrmann S, Dernoncourt F, Li Y, Carlson ET, Wu JT, Welt J, et al Comparing deep learning and concept extraction based methods for patient phenotyping from clinical narratives. PLoS ONE. 2018;13:e0192360 10.1371/journal.pone.0192360 29447188 10.1371/journal.pone.0192360 PMC5813927

[9] Shah SJ, Katz DH, Selvaraj S, Burke MA, Yancy CW, Gheorghiade M, et al Phenomapping for novel classification of heart failure with preserved ejection fraction. Circulation. 2014;131:269–79. 10.1161/CIRCULATIONAHA.114.010637 25398313 10.1161/CIRCULATIONAHA.114.010637 PMC4302027

[10] Rajkomar A, Oren E, Chen K, Dai AM, Hajaj N, Liu PJ, et al Scalable and accurate deep learning for electronic health records. NPJ Digit Med. 2018;1:18 10.1038/s41746-018-0029-1 10.1038/s41746-018-0029-1 PMC6550175 31304302

[11] Zhang J, Gajjala S, Agrawal P, et al Fully automated echocardiogram interpretation in clinical practice. Circulation. 2018;138:1623–35. 10.1161/CIRCULATIONAHA.118.034338 30354459 10.1161/CIRCULATIONAHA.118.034338 PMC6200386

[12] Douglas PS, De Bruyne B, Pontone G, Patel MR, Norgaard BL, Byrne RA, et al 1-year outcomes of FFRCT-guided care in patients with suspected coronary disease: the PLATFORM study. J Am Coll Cardiol. 2016;68:435–45. 10.1016/j.jacc.2016.05.057 27470449 10.1016/j.jacc.2016.05.057

[13] Mortazavi BJ, Downing NS, Bucholz EM, Dharmarajan K, Manhapra A, Li S, et al Analysis of machine learning techniques for heart failure readmissions. Circ Cardiovasc Qual Outcomes. 2016;9:629–40. 10.1161/CIRCOUTCOMES.116.003039 28263938 10.1161/CIRCOUTCOMES.116.003039 PMC5459389

[14] Char DS, Shah NH, Magnus D. Implementing machine learning in health care—addressing ethical challenges. N Engl J Med. 2018;378:981–3. 10.1056/NEJMp1714229 29539284 10.1056/NEJMp1714229 PMC5962261

[15] Australian and New Zealand Intensive Care Society Centre for Outcome and Resource Evaluation. ANZICS CORE annual report 2017 Melbourne: Australian and New Zealand Intensive Care Society Centre for Outcome and Resource Evaluation; 2017.

[16] Salter R, Bailey M, Bellomo R, Eastwood G, Goodwin A, Nielsen N, et al Changes in temperature management of cardiac arrest patients following publication of the Target Temperature Management Trial. Crit Care Med. 2018;;46:1722–30. 10.1097/CCM.0000000000003339 30063490 10.1097/CCM.0000000000003339

[17] Performance of APACHE III over time in Australia and New Zealand: a retrospective cohort study. Anaesth Int Care. 2012;40:980–94. 10.1177/0310057X1204000609 23194207

[18] Pilcher D, Paul E, Bailey M, Huckson S. The Australian and New Zealand Risk of Death (ANZROD) model: getting mortality prediction right for intensive care units. Crit Care Resusc. 2014;16:3–4. 10.1016/j.jcrc.2013.07.058.2 24588429

[19] Hastie T, Tibshirani R, Friedman J. The elements of statistical learning Springer Series in Statistics. New York: Springer; 2017 10.1007/b94608

[20] LeCun Y, Bengio Y, Hinton G. Deep learning. Nature. 2015;521:436–44. 10.1038/nature14539 26017442 10.1038/nature14539

[21] Brier GW. Verification of forecasts expressed in terms of probability. Mon Weather Rev. 1950;78:1–3. 10.1175/1520-0493(1950)078<0001:VOFEIT>2.0.CO;2

[22] Rufibach K. Use of Brier score to assess binary predictions. J Clin Epidemiol. 2010;63:938–9. 10.1016/j.jclinepi.2009.11.009 20189763 10.1016/j.jclinepi.2009.11.009

[23] Ribeiro MT, Singh S, Guestrin C. Why should i trust you? Explaining the predictions of any classifier In: Proceedings of the 22nd ACM SIGKDD International Conference on Knowledge Discovery and Data Mining. New York: ACM; 2016 pp. 1135–44. 10.1145/2939672.2939778

[24] Pedregosa F, Varoquaux G, Gramfort A, Michel V, Thirion B, Grisel O, et al Scikit-learn: machine learning in Python. J Mach Learn Res. 2011;12:2825–30.

[25] McKinney W. Data structures for statistical computing in Python. In: Proceedings of the 9th Python in Science Conference (SciPy 2010). SciPy; 2010. pp. 51–56.

[26] R Core Team. R: a language and environment for statistical computing Version 3.4.4. Vienna: R Foundation for Statistical Computing; 2018.

[27] Robin X, Turck N, Hainard A, Tiberti N, Lisacek F, Sanchez JC, et al pROC: an open-source package for R and S+ to analyze and compare ROC curves. BMC Bioinformatics. 2011;12:77 10.1186/1471-2105-12-77 21414208 10.1186/1471-2105-12-77 PMC3068975

[28] Wickham H. Tidyverse: easily install and load the ‘Tidyverse’ Version 1.2.1. Vienna: R Foundation for Statistical Computing; 2017 [cited 2018 Nov 8]. Available from: https://CRAN.R-project.org/package=tidyverse/.

[29] Adrie C, Cariou A, Mourvillier B, Laurent I, Dabbane H, Hantala F, et al Predicting survival with good neurological recovery at hospital admission after successful resuscitation of out-of-hospital cardiac arrest: the OHCA score. Eur Heart J. 2006;27:2840–5. 10.1093/eurheartj/ehl335 17082207 10.1093/eurheartj/ehl335

[30] Aschauer S, Dorffner G, Sterz F, Erdogmus A, Laggner A. A prediction tool for initial out-of-hospital cardiac arrest survivors. Resuscitation. 2014;85:1225–31. 10.1016/j.resuscitation.2014.06.007 24960427 10.1016/j.resuscitation.2014.06.007

[31] Huang C-H, Tsai M-S, Chien K-L, Chang W-T, Wang T-D, Chen S-C, et al Predicting the outcomes for out-of-hospital cardiac arrest patients using multiple biomarkers and suspension microarray assays. Sci Rep. 2016;6:27187 10.1038/srep27187 27256246 10.1038/srep27187 PMC4891702

[32] Lundberg S, Lee S-I. A unified approach to interpreting model predictions. arXiv:1705.07874v2. arXiv; 2017 11 25.

[33] Silva I, Moody G, Scott D. Predicting in-hospital mortality of ICU patients: the PhysioNet/Computing in Cardiology Challenge 2012. Comput Cardiol (2010). 2012;5:245–8. PMC3965265 24678516

