# Supplementary figures and images for: Characterising risk of in-hospital mortality following cardiac arrest using machine learning: A retrospective international registry study
Source: PLoS Med. 2018 Nov 30;15(11):e1002709. doi: 10.1371/journal.pmed.1002709 (PMC6267953; doi:10.1371/journal.pmed.1002709)

**S1 Fig.** Calibration plots for all models.

| 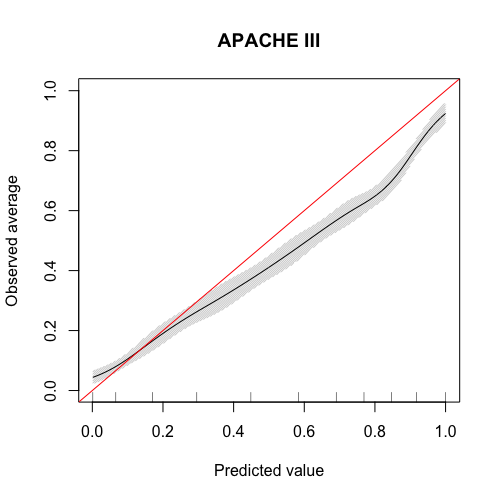 | 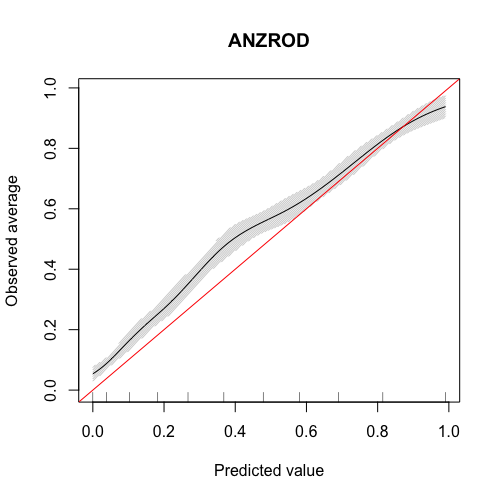 |
| --- | --- |
| 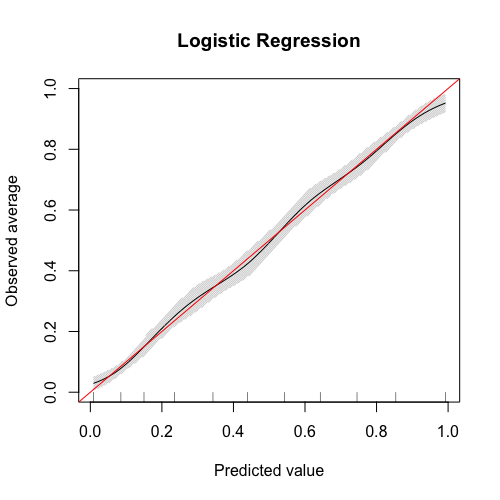 | 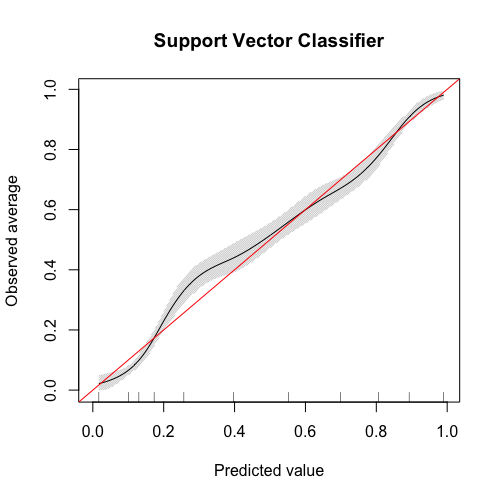 |
| 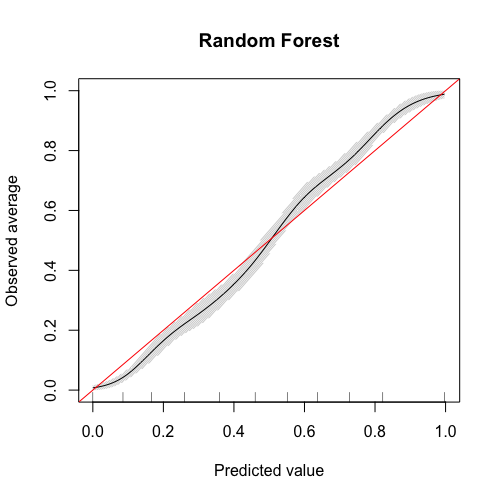 | 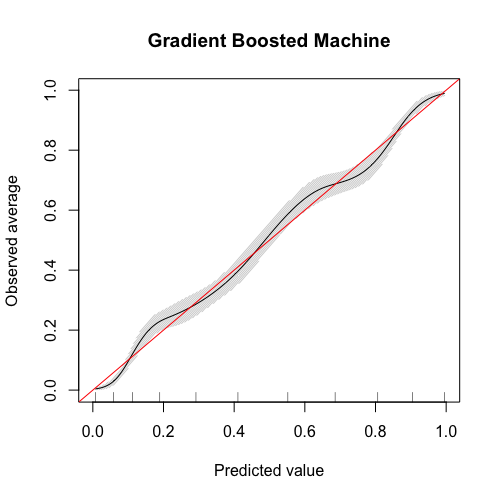 |
| 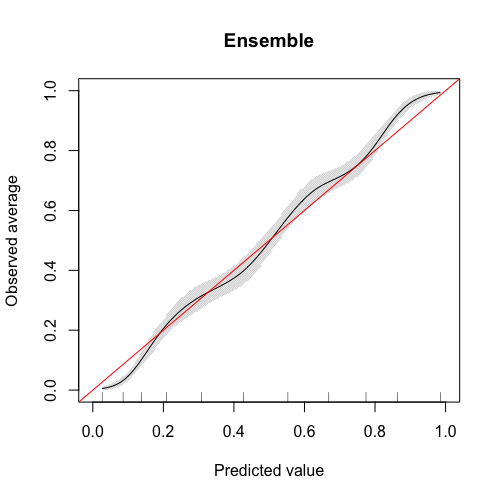 | 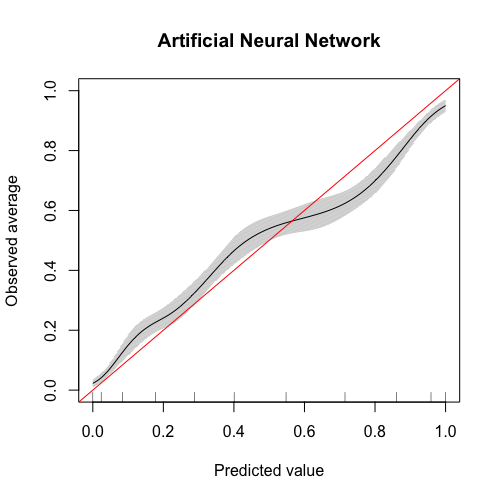 |

Supplement: S1 Fig — (DOCX) [file pmed.1002709.s001.docx]

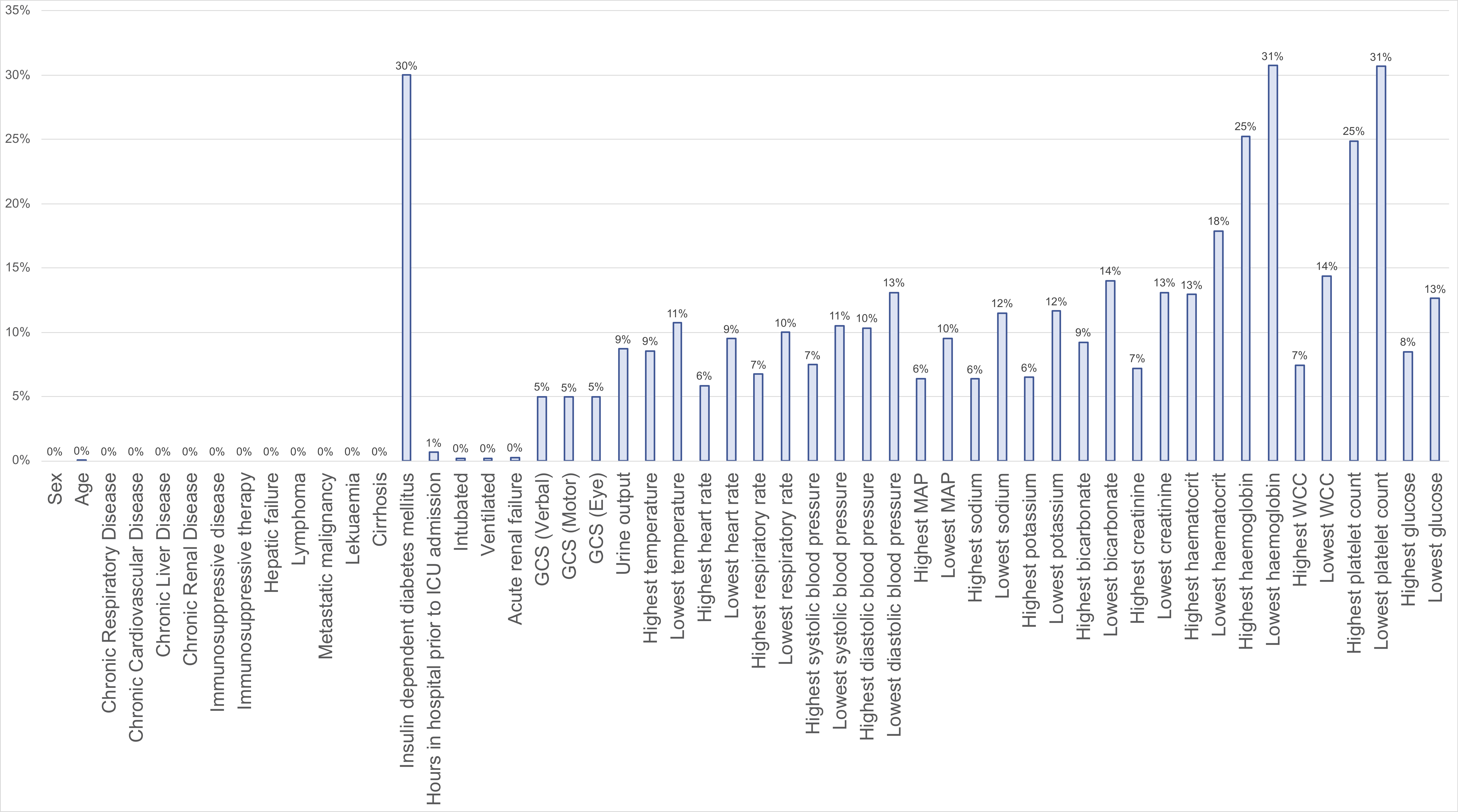

Supplement: S2 Fig — (PNG) [file pmed.1002709.s002.png]
