# Supplementary material for: Characterising risk of in-hospital mortality following cardiac arrest using machine learning: A retrospective international registry study
Source: PLoS Med. 2018 Nov 30;15(11):e1002709. doi: 10.1371/journal.pmed.1002709 (PMC6267953; doi:10.1371/journal.pmed.1002709)
Supplement: S1 Table — (DOCX) [file pmed.1002709.s003.docx]

| S1 Table. Variables included in the logistic regression and ML models.  Age |
| --- |
| Sex |
| Individual components (verbal, motor, eye) of the Glasgow Coma Score |
| Intubated or ventilated |
| Urine output |
| Hours in hospital prior to intensive care unit admission |
| CHRONIC MEDICAL CONDITIONS |
| Chronic respiratory disease |
| Chronic cardiovascular disease |
| Chronic liver disease, cirrhosis or hepatic failure |
| Acute renal failure or chronic renal disease |
| Immunosuppressive disease |
| Immunosuppressive therapy, including high dose steroids |
| Lymphoma |
| Metastatic malignancy |
| Leukaemia |
| Insulin dependent diabetes mellitus |
| HIGHEST AND LOWEST PHYSIOLOGIC/BIOCHEMICAL MEASURES |
| Temperature  Heart Rate  Respiratory Rate  Systolic Blood Pressure  Diastolic Blood Pressure  Mean Arterial Pressure  Sodium  Potassium  Bicarbonate  Creatinine  Haematocrit  Haemoglobin  White Cell Count  Platelet Count  Glucose |
