# Supplementary material for: Characterising risk of in-hospital mortality following cardiac arrest using machine learning: A retrospective international registry study
Source: PLoS Med. 2018 Nov 30;15(11):e1002709. doi: 10.1371/journal.pmed.1002709 (PMC6267953; doi:10.1371/journal.pmed.1002709)
Supplement: S2 Table — (DOCX) [file pmed.1002709.s004.docx]

**S2 Table. Comparison of performance metrics calculated on the test set, based on optimal thresholds (maximising sensitivity and specificity) obtained from receiver operating characteristic curves.** Sens, sensitivity; Spec; specificity, PPV, positive predictive value; NPV, negative predictive value; Prec, precision; Rec, recall; Acc, accuracy.

| Model | AUC (95% CI) | Thresh. | Sens. | Spec. | PPV | NPV | Prec. | Rec. | Acc. |
| --- | --- | --- | --- | --- | --- | --- | --- | --- | --- |
| APACHE III risk of death | 0.80 (0.79-0.82) | 0.56 | 0.69 | 0.69 | 0.67 | 0.79 | 0.67 | 0.77 | 0.73 |
| ANZROD | 0.81 (0.80-0.82) | 0.37 | 0.75 | 0.71 | 0.69 | 0.78 | 0.69 | 0.75 | 0.73 |
| Logistic Regression | 0.82 (0.81-0.83) | 0.50 | 0.72 | 0.79 | 0.74 | 0.77 | 0.74 | 0.72 | 0.75 |
| Artificial Neural Network | 0.85 (0.84-0.86) | 0.37 | 0.82 | 0.72 | 0.71 | 0.83 | 0.71 | 0.82 | 0.77 |
| Random Forest | 0.86 (0.84-0.87) | 0.5 | 0.76 | 0.79 | 0.75 | 0.80 | 0.75 | 0.76 | 0.78 |
| Support vector classifier | 0.86 (0.85-0.87) | 0.49 | 0.75 | 0.81 | 0.77 | 0.79 | 0.77 | 0.75 | 0.78 |
| Ensemble | 0.87 (0.86-0.88) | 0.49 | 0.77 | 0.81 | 0.77 | 0.81 | 0.77 | 0.77 | 0.79 |
| Gradient boosted machine | 0.87 (0.86-0.88) | 0.44 | 0.80 | 0.78 | 0.75 | 0.82 | 0.75 | 0.79 | 0.79 |
